# Epitaxy of highly ordered organic semiconductor crystallite networks supported by hexagonal boron nitride

**DOI:** 10.1038/srep38519

**Published:** 2016-12-08

**Authors:** Aleksandar Matković, Jakob Genser, Daniel Lüftner, Markus Kratzer, Radoš Gajić, Peter Puschnig, Christian Teichert

**Affiliations:** 1 Institute of Physics, Montanuniversität Leoben, Franz Josef Strasse 18, Leoben, 8700, Austria; 2 Institute of Physics, Karl-Franzens-Universität Graz, NAWI Graz, Universitätsplatz 5, Graz, 8010, Austria; 3 Graphene Laboratory (GLAB) of Center for Solid State Physics and New Materials, Institute of Physics, University of Belgrade, Pregrevica 118, Belgrade, 11080, Serbia

## Abstract

This study focuses on hexagonal boron nitride as an ultra-thin van der Waals dielectric substrate for the epitaxial growth of highly ordered crystalline networks of the organic semiconductor parahexaphenyl. Atomic force microscopy based morphology analysis combined with density functional theory simulations reveal their epitaxial relation. As a consequence, needle-like crystallites of parahexaphenyl grow with their long axes oriented five degrees off the hexagonal boron nitride zigzag directions. In addition, by tuning the deposition temperature and the thickness of hexagonal boron nitride, ordered networks of needle-like crystallites as long as several tens of micrometers can be obtained. A deeper understanding of the organic crystallites growth and ordering at ultra-thin van der Waals dielectric substrates will lead to grain boundary-free organic field effect devices, limited only by the intrinsic properties of the organic semiconductors.

For organic semiconductor electronics^1^ adequate interfaces with dielectric, metallic, and inorganic semiconducting layers are required to reach its full potential. Interface engineering has been demonstrated to be an effective approach to increase the quality of organic semiconductor (OSC) thin films, enabling significant enhancements in performance of organic field effect transistors (OFETs)^2^,^3^. By tuning the properties of the organic-inorganic interfaces in OFETs, it is possible to obtain thin-film devices^4^,^5^ whose performance can compete with OSC single-crystal, air- and vacuum-gap devices^2^,^3^.

Recently, two-dimensional (2D) van der Waals (vdW) materials^6^ have been proposed as substrates for the epitaxy of organic molecules^5^,^7^,^8^,^9^,^10^,^11^,^12^. Their key advantage lies in the fact that these interfaces have vdW nature^13^, meaning that surfaces on which OSCs grow are atomically smooth with no dangling bonds and trapped charges at the interface. This enables growth of high-quality organic crystal thin films with very large grains and low amount of defects^5^. Heterointerfaces with vdW gap between OSCs and 2D materials have been first utilized employing graphene as a carrier injection layer between OSCs and metallic contacts^7^, and as vdW electrodes^14^. Besides graphene, layered semiconductors such as transition metal dichalcogenides have been used to form vdW heterostructures with OSCs^9^,^15^, even realizing atomically thin vdW p-n junctions^11^.

Probably, the most important interface in OFETs is the gate dielectric/OSC interface. It defines transport, since carriers accumulate at the interface only in the first few molecular layers of the OSC^16^. The gate dielectric’s surface energy^17^ and roughness^18^ affect the growth of OSCs, thus increasing grain boundary density and consequently reducing carrier transport in OFETs^19^. Furthermore, the gate dielectric/OSC interface has a major impact on the device stability through surface trap density^20^, organic layer morphology, and aggregation^3^,^21^. Growth of highly ordered organic crystal thin-films on conventional dielectric substrates such as SiO_2_ and Al_2_O_3_ proves to be very challenging^3^. Tuning the gate dielectric surface by various treatments such as using self assembling mono-layers or polymers as gate dielectrics show significant enhancement of the OFETs electrical properties^19^,^20^. On the other hand, limited access to vdW dielectric materials has restrained their wider application in OSC devices. Recent advances in growth of single crystal hexagonal boron nitride (hBN)^22^ together with micro-mechanical exfoliation technique, as demonstrated for graphene^23^ and other 2D materials^6^, enable fabrication of high-quality atomically flat hBN thin-films.

hBN has great potential to be used as a gate material in field-effect devices, being an almost defect-free dielectric with a breakdown field in the order of 1 GV/m^24^. However, atomically thin flakes would most likely not be suitable for FET applications, since electrons can tunnel through very thin hBN^24^. On the other hand, single layer hBN has been employed for encapsulation of other 2D materials, thus allowing for top electrical contacts, and at the same time preventing degradation of the encapsulated material upon exposure to ambient conditions^25^.

As a vdW dielectric substrate, hBN has been employed to support graphene^26^, reducing interface roughness, improving stability and intrinsic local electronic properties, compared to graphene supported by commonly used SiO_2_/Si. hBN has also been used as a gate dielectric in graphene field-effect devices^27^,^28^, and recently to encapsulate black phosphorous and niobium diselenide devices^25^,^29^. Highly crystalline thin-films of pentacene supported by hBN have allowed for probing of intrinsic charge transport down to a monolayer^5^. Furthermore, hBN was employed as a vdW gate dielectric in top-gated rubrene single crystal OFETs^30^. Epitaxial growth of rubrene on exfoliated hBN was demonstrated^10^, showing that the electrical properties of these devices approach those of single crystal ones. In order to fully exploit heterostructures between vdW materials and OSCs, a deeper understanding of their interface is essential. While pentacene^5^ and rubrene^10^ grow in a layer-by-layer mode on hBN, an interesting advantage for many applications, especially in organic optoelectronic, would be to have essentially one-dimensional (1D) crystallites grown with high degree of ordering, thus enabling for example highly polarized light emission.

In this study, we examine hBN as a vdW dielectric substrate for epitaxial thin-film growth of one type of rod-like conjugated molecule. For this purpose, we use hot wall epitaxy (HWE)^31^ deposition of parahexaphenyl (6P), serving as a model system for the growth of organic semiconductors^32^ which has been recently applied to detect cleanliness of poly(methyl methacrylate) assisted wet transfer of chemical vapor deposited graphene^33^. Using atomic force microscopy (AFM), we investigate how the thickness of the supporting hBN and the deposition temperature affect the morphology of the obtained 6P thin-films. We show that, by tuning the growth parameters, needles as long as several tens of micrometers can be obtained on hBN support. In particular, we investigate how the symmetry of the hBN substrate is reflected by the growth directions of the needle-like 6P crystallites. These are related to the preferred adsorption sites for individual molecules that we calculate using density functional theory (DFT).

## Results and Discussion

The results are divided into three subsections. First, the results related to the epitaxial relation and the contact plane between 6P and hBN, as well as the growth directions of the crystallites, are considered. In the second subsection, the effects that the number of hBN layers has on the morphology of the grown crystallites are shown. The final subsection addresses the effects of the deposition temperature (T_*D*_) on the crystallite size, and the total 6P volume on hBN.

### Contact plane and angular distribution of 6P needles on hBN

In this subsection we focus on how the vdW interface, and the substrate’s symmetry affect the self-assembly of 6P needle networks on hBN. Note that a similar formation of 6P needle-like crystallite networks was observed on KCl^34^ and mica^35^. Having a well ordered network of needle-like crystallites is essential for many applications, such as high mobility devices, sensors, polarized emission, optical resonators, waveguides, or lasing devices^36^,^37^. A major contributor to self-assembly of crystallite networks is the molecule-substrate interface. In the case of hBN and other 2D materials, on which organic crystals grow through vdW epitaxy, the substrate symmetry plays a major role since the sample and the substrate are rotationally commensurate in spite of the lattice constant mismatch^13^.

**Figure 1:**
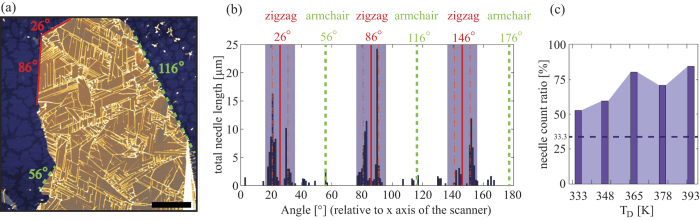
(**a**) 20 × 20 *μ*m^2^ AFM image of 18.1 nm thick bulk hBN flake covered with 6P needles (T_*D*_ = 363 K, lateral scale bar 4 *μ*m, logarithmic z scale 65 nm). (**b**) Total needle length in a particular direction with ±0.5° tolerance, considering x axis of the AFM scanner as 0°. Solid red lines and dashed green lines in (**a**) and (**b**) indicate zigzag and armchair edges of the hBN flake, respectively (the procedure to index flake edges is presented at the end of the first subsection). Dot-dashed red lines in (**b**) represent ±5° splitting from zigzag directions of hBN flakes. (**c**) The fraction of needles that are in the preferred growth directions, as a function of T_*D*_. The preferred growth directions are marked as shaded areas in (**b**). The dashed line set to 33.3% corresponds to the case of a completely disordered network.

For the analysis of the self-assembled needle networks, the angular distribution of 6P needles on bulk hBN flakes was evaluated. Figure 1(a) shows an AFM topography image of the 6P needle network, grown on a 18.1 nm thick hBN flake (T_*D*_ = 363 K). In order to obtain the angular distribution of the needles, the direction of each needle was considered with respect to the *x* axis of the scanner, and measured counterclockwise. The total length of all needles in Fig. 1(a) that are aligned in a particular direction with a ±0.5° tolerance is presented in 1(b). Also, the directions of sharp edges of the hBN flake are shown in Fig. 1(a,b), and are denoted by solid-red and dashed-green lines. These are used to correlate the growth directions of 6P with the potential high-symmetry directions of the hBN flake. On most of the hBN flakes used in this study, the directions of the sharp flake edges were separated by an integer multiplier of 30°, thus indicating that these correspond to armchair and zigzag directions of the hBN. Interestingly, as it can be seen from Fig. 1(b), 6P needles tend to follow primarily one type of these directions, namely the ones marked by solid-red lines. The first question to raise is in which of the high-symmetry directions the needles grow, armchair or zigzag? And the second question is about the origin of the ±5° deviation from this direction. Both points can be addressed considering the epitaxial relation between bulk 6P and the hBN basal plane. Previously reported results for epitaxy of graphene^38^ and pentacene^5^ on hBN would indicate that individual molecules tend to adsorb face-on with the molecular long axis parallel to armchair direction of hBN. To prove this statement, we have performed first-principle calculations within the framework of DFT.

**Figure 2:**
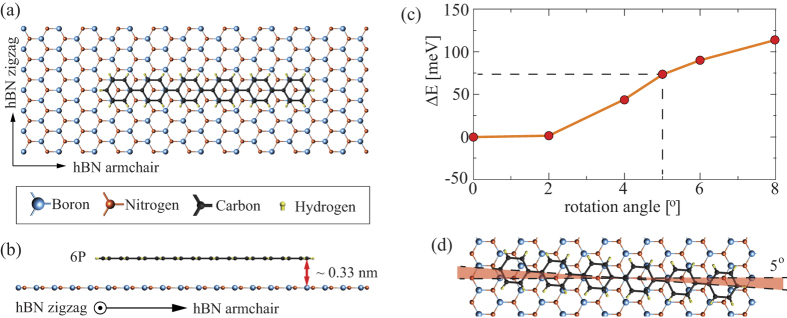
Scheme of the preferred adsorption site for an isolated 6P molecule on a single layer hBN as obtained by DFT calculations, (**a**) top view, (**b**) side view. The molecule prefers to be face-on, oriented along armchair direction with the center of the phenyl rings above N and an adsorption height of about 0.33 nm. (**c**) A relative change of the adsorption energy with a slight rotation of 6P molecule from the preferred adsorption site. (**d**) Schematic representation of the rotated molecule for the case of 5° angle.

We have calculated the adsorption energies of single 6P molecules on one layer hBN substrate for nine different adsorption sites with the long axis of the 6P molecules oriented along the armchair direction of hBN, and four adsorptions sites with 6P oriented along the zigzag direction. For all of them, we have optimized the internal atomic coordinates and found an average distance between 6P and the hBN substrate of about 0.33 nm. Among all these structures, the preferred adsorption site is the one with the center of the phenyl rings above the N atom of the hBN substrate and 6P’s long axis oriented along the armchair direction. The adsorption energy for this site is about 170 meV larger than the most stable site with 6P oriented along the zigzag direction. Top and side views of the most favorable structure are depicted in Fig. 2(a) and (b), respectively. Note that for pentacene on hBN, a similar adsorption site has been found, with the phenyl rings above N. However, pentacene aligns along the zigzag direction^5^, since it may be regarded as a snippet of graphene in this direction^39^.

Taking into account the preferred orientation of a single 6P molecule (as presented in Fig. 2(a)) suggests that during the initial stages of the growth deposited molecules attach in the direction perpendicular to the long axis of the molecule, that is along the zigzag directions of the hBN substrate. In order to clarify the origin of the ±5° splitting in the needle growth directions, we have further investigated with DFT whether this small deviation can be explained by a slight rotation of the individual molecules. The energy dependence on the rotational angle is shown in Fig. 2(c) and demonstrates that a misalignment of about 5°, as presented in Fig. 2(d), results in a significant increase in adsorption energy and thus can be ruled out as an origin for the deviation of the observed needle orientations from the zigzag direction of hBN. An alternative explanation for the observed slight deviation of the needle’s long axis from the high-symmetry direction of the hBN can be found considering the epitaxial relation between 6P crystallites and supporting hBN. This will be elucidated in the following.

As mentioned above, the preferred adsorption site of individual molecules provides only a starting point for the alignment of 6P crystals on hBN, during the initial stages of the growth. While the density of the molecules on the surface of hBN is small enough, substrate-molecule interaction dominates and the molecules tend to adopt their individual equilibrium positions. However, once a critical density is reached, intermolecular interaction takes over and molecules reorganize mainly into the equilibrium bulk structure^36^. At that point, the contact plane of the bulk molecular crystal which introduces the least amount of strain between the equilibrium adsorption sites of individual molecules and the bulk structure is chosen, being energetically most favored. In the case presented here, 6P crystallites are large enough to be safely considered as bulk, and in the considered range of T_*D*_ only the monoclinic *β*-phase of 6P crystallites is expected (the Baker structure)^40^. Furthermore, if the preferred adsorption sites of the individual molecules are taken into account, the long axis of the molecules can be fixed to an armchair direction of hBN. Then, considering all known contact planes of 6P needle-like crystallites with single crystal substrates^36^, only the 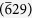 plane (previously reported for Cu (110) surface^41^) can describe the splitting of needle growth directions with respect to the zigzag directions of hBN.

**Figure 3:**
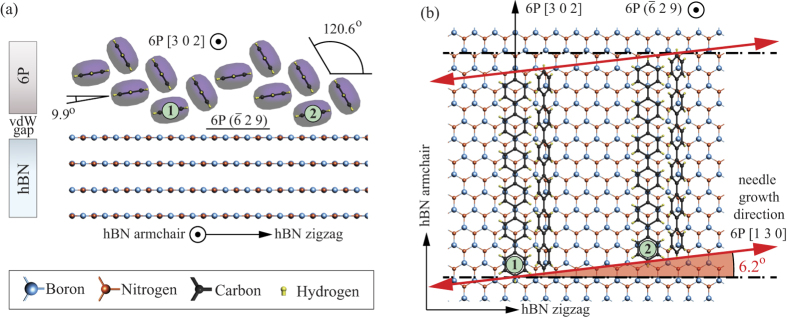
A schematic presentation of the 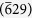 6P contact plane with hBN basal plane, (**a**) side view of the interface, and (**b**) top view. Molecules closest to the hBN surface are labeled with the numbers 1 and 2. Red arrows in (**b**) represent the 6P [130] direction that corresponds to the long axis of the resulting 6P needles.

Figure 3 shows a schematic representation of the 6P *β*-phase with the 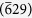 plane in contact with the hBN (0001) plane. Molecules labeled with the numbers 1 and 2 are closest to the hBN surface, and have their phenyl ring plane nearly parallel (tilted by 9.9°) to the hBN (0001) plane. These molecules will tend to assume the positions as close as possible to the equilibrium adsorption sites of individual molecules. Interestingly, if one molecule is fixed at the preferred adsorption site, all other molecules that are in contact with an hBN plane will fall into equivalent sites, related only by translational symmetry of hBN. This further confirms that 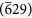 contact plane optimizes between a bulk Baker structure of 6P and the preferred adsorption sites of the individual molecules on hBN. As indicated by the red arrows in Fig. 3(b), this particular epitaxial relation of hBN and 6P clearly explains the distinct split of the needle long axis orientation with respect to the hBN zigzag direction. Considering the three-fold rotation and mirror symmetries of hBN gives a total of six preferred growth directions of 6P needle-like crystallites (as shown in Fig. 1(b)). These self-assembled crystallite networks can be used to unambiguously determine the crystallographic orientation of the underlying hBN, including the identification of the edge type (as has been done in retrospective in Fig. 1(a)). Such a determination procedure does not require special preparation of hBN flakes, or a large surface area, it does not rely on any elaborate measuring techniques, and neither requires atomic nor nanometer-scale resolution. In other words, it would be even sufficient to inspect the samples under the optical microscope where larger 6P crystallites are visible.

The above described self-assembly of the 6P needle networks was observed for all the samples in the entire range of T_*D*_ considered in this study. In order to quantify the degree of ordering of the crystallite networks, the range of ±10° from the zigzag direction of the hBN support was defined as the preferred growth direction range for the needles (shaded areas in Fig. 1(b)). Considering three-fold symmetry of the hBN support, one third of all possible growth directions are consequently considered as preferred. Figure 1(c) presents the evolution of the preferential growth directions of 6P needles with respect to T_*D*_. The results show that with an increase of T_*D*_, more and more needles tend to grow in the preferred directions, indicating that these directions are indeed energetically the most favorable ones. Note that the dashed line at 33.3% in Fig. 1(c) represents the case of a completely disordered crystallite network, while the values below this line would indicate that the chosen directions are not favorable. Further results related to the influence of T_*D*_ on the crystallite growth are presented in the third subsection.

### Effects of hBN thickness on the morphology of 6P crystallites

As mentioned in the introduction, hBN has great potential to serve as a flexible gate dielectric material, since it is chemically and mechanically stable, and even ultra-thin films can sustain very high electric fields^24^,^25^. In this subsection, we will focus on how the thickness of the hBN support affects the morphology of 6P crystallites. For this purpose, the deposition temperature has been fixed at T_*D*_ = 363 K. At this specific T_*D*_, the nucleation density is high enough such that the inspected 10 × 10 *μ*m^2^ sections of the substrate area allow for statistical analysis of 6P crystallites. At higher T_*D*_, the dependence of the 6P morphology on the thickness of hBN is expected to be even further enhanced, however, a larger substrate area would be needed for a proper statistical analysis of crystallite lengths and orientations. Furthermore, at higher T_*D*_, a significant amount of needles is terminated at the flake edges, especially in the case of the smallest and the thinnest flakes. This would certainly affect the results of the measured needle lengths. At 363 K (and at lower temperatures), the majority (over 80%) of the needles are not terminated by the flake edges. The same trend for the dependence of the morphology of 6P crystallites with respect to thickness of the hBN support was observed in the entire range of T_*D*_.

**Figure 4:**
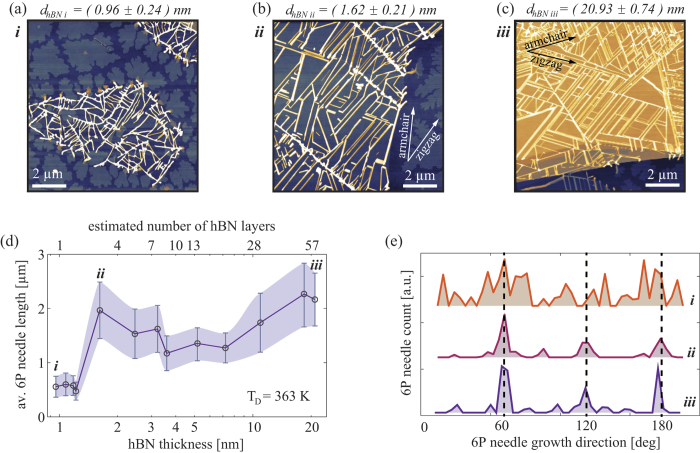
The influence of hBN substrate thickness on 6P needle length and orientation, at T_*D*_ = 363 K. (**a**–**c**) 10 × 10 *μ*m^2^ AFM topography images of 6P crystallite on hBN flakes with varied hBN thickness. Logarithmic z scales are 15 nm (**a**,**b**), and 30 nm (**c**). The arrows in (**b**) and (**c**) indicate high-symmetry directions of the supporting hBN flakes. (**d**) The dependence of the average 6P needle length on hBN thickness, each point in (**d**) represents a different hBN flake and over 100 needles were considered on each flake. Points denoted in (**d**) by *i, ii*, and *iii* are further analyzed in (**e**) with respect to the needle orientation, and are respectively shown in (**a**–**c**). (**e**) Angular distribution of 6P needles for the three selected hBN thicknesses. The *x*-axis in (**e**) is normalized for each sample to align the zigzag directions of supporting hBN flakes (dashed vertical lines).

Figure 4 shows how the thickness of the hBN support affects the average length and degree of ordering of 6P needles. We observe an interesting effect: hBN flakes that are less than ~1.5 nm thick (estimated as 1–2 layer flakes) have an average needle length of 0.6 *μ*m (see Fig. 4(a)), while hBN flakes that are thicker than 1.5 nm have over 3 times longer needles (Fig. 4(b)). The average length of 6P needles was not found to be increasing with further increase of the hBN thickness, and even beyond 20 nm hBN thickness, very similar morphology of 6P needles as for 1.6 nm thick flakes was observed (Fig. 4(c)). The average height of the needles (in this case ~12 nm) was found not to be affected by the thickness of the hBN substrate. Figure 4(d) presents the relation between hBN thickness and an average length of 6P needles, here each point represents a different hBN flake and over 100 needles were considered on each flake. Besides needle-like crystallites that grow on hBN, in Fig. 4(a) island-like crystallites (composed of up-right standing molecules) are observed on the surrounding SiO_2_ surface. More details related to orientation of 6P molecules and growth of crystallites is given in the Methods section.

Besides the average length of the needles, their angular distribution is also affected by the thickness of the hBN support. Figure 4(e) shows the angular distribution of 6P needles (with ±2.5° tolerance) on top of three different hBN flakes, presented in Fig. 4(a–c), and denoted in Fig. 4(d) with *i, ii*, and *iii*. The *x*-axis in Fig. 4(e) is normalized for each sample with respect to the high-symmetry directions of the supporting hBN flakes. The directions indicated by dashed vertical lines reflect the three-fold rotational symmetry of the underlaying hBN, and show zigzag directions. Splitting of the preferred growth directions of 6P crystallites from zigzag directions of hBN is not visible in this case due to larger histogram intervals (5°), and the fact that smaller surface areas were analysed. The samples supported by 1–2 hBN layers do not exhibit preferential orientation of 6P needles, while samples supported by few-layer and bulk hBN have needles that follow zigzag directions of the supporting flakes.

In order to understand the experimentally observed dependence of the needle orientation on the hBN thickness and to check whether there is a direct influence of the thickness onto the 6P needle growth, we have performed calculations of 6P on one and three layers of hBN, respectively. For the three-layer case, the same lateral size of the cell was used, and two additional hBN layers have been placed underneath the first one in AA′ stacking configuration. We have considered two alignments of the 6P molecule, one with the long axis oriented along the armchair direction and one along the zigzag direction of the substrate. For each orientation, we considered the most stable adsorption site, as determined for 6P on a single layer of hBN, and calculated the respective adsorption energies. Because the so calculated adsorption energies do not show any dependence on the thickness of the hBN substrate, we conclude that another, indirect mechanism causes the experimentally observed layer dependence.

The observed abrupt change in the morphology of 6P needles with an increase of hBN thickness can be explained by two contributing factors that arise from the hBN/SiO_2_ interface. First, hBN reduces the influence of the surface roughness of underlying SiO_2_^27^, and the top surface of hBN becomes less rough the more hBN layers are added. A higher surface roughness is well known to introduce disorder in OSC thin-films^18^, and this is most likely the case here for thin hBN flakes. The surface roughness of hBN was estimated as an average root-mean-square (RMS) value taken from ten 1 × 1 *μ*m^2^ areas of each flake prior to the deposition of the molecules. The RMS surface roughness of the surrounding SiO_2_ area was found to be (0.23 ± 0.03) nm, whereas it was slightly smaller (0.21 ± 0.03) nm for very thin flakes (less than 1.5 nm thick). However, for few-layer hBN flakes it was significantly reduced to (0.15 ± 0.03) nm. The obtained RMS roughness values are in good agreement with the data reported in the literature^27^. It is worth mentioning that the reduction in the RMS from SiO_2_ to hBN was smaller in our study, since dry thermal oxidation of silicon was used resulting in a smoother SiO_2_ surface. Although in our case the surface roughness of thin hBN flakes is higher than that of the few-layer ones, we still expect that this is not the only contributing factor to the observed differences in 6P morphology on thin and thick hBN flakes.

The other likely contributing factor to the observed difference in 6P morphology on thin and few-layer hBN flakes could be related to the hBN/SiO_2_ interface, since hBN is deposited by mechanical exfoliation a trapped water layer is expected at the interface with SiO_2_, giving rise to a dipole field. This effect was previously observed by electrostatic force microscopy^42^. Since hBN is an insulator, additional layers will not be very effective in screening of this dipole field^42^, and the field will decay relatively slowly with additional hBN layers.

The effects of a water layer induced dipole field on the growth of OSCs were demonstrated for pentacene grown on graphene^43^, where much stronger dielectric screening is present^44^. There, it was found that a trapped water layer significantly affects the growth of pentacene on graphene, changing from upright standing islands when a water layer is present, to needles when this layer is suppressed^43^. Interestingly, for 6P on graphene an opposite tendency for needle growth was detected with an increase of supporting flake thickness^45^, and was attributed to the changes in surface energy of graphene with additional layers. Here, in the case of hBN supported 6P, we expect that both, surface roughness of hBN and the unscreened dipole field from trapped water layer are responsible for the different morphologies of 6P on thin and few-layer hBN flakes. Thus, a critical thickness of ~1.5 nm for hBN support is needed to obtain more ordered networks of longer crystallites.

### Effects of the deposition temperature on the growth of 6P on hBN

As has already been presented in Fig. 1(c), the deposition temperature (T_*D*_) has a major impact on the resulting ordering of 6P crystallites grown on hBN. In addition, according to classical nucleation theory, by increasing T_*D*_, the diffusion of the molecules on the surface is increased and the nucleation density is reduced^46^. As a result, larger single crystal grains are obtained, thus reducing the number of grain boundaries per unit area. In the deposition temperature range considered in this study (300–400 K), both islands and needles were found to grow on hBN. However, the majority of 6P (over 95% on average) forms needle-like crystallites in the considered range of T_*D*_. For this reason, we focus here on how T_*D*_ affects the growth of 6P needles.

**Figure 5:**
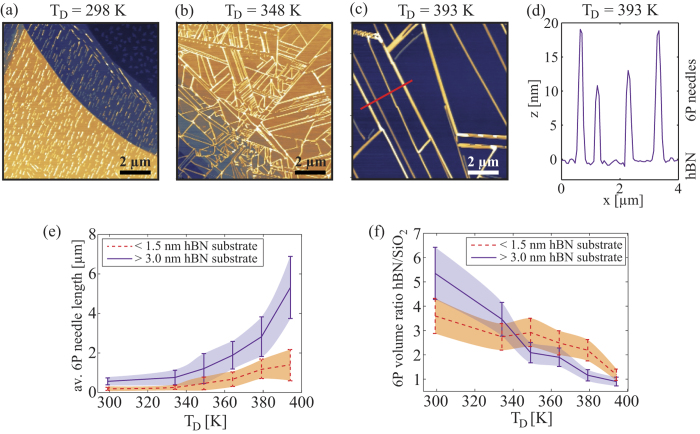
(**a**–**c**) AFM topography images showing the dependence of 6P morphology with increasing T_*D*_. All images show 10 × 10 *μ*m^2^ areas of bulk hBN flakes (logarithmic z scales: 50 nm (**a**), 25 nm (**b**,**c**)). (**d**) Shows the height profile of the red line denoted in (**c**), which intersects several 6P needles. The average length of 6P needles (**e**), and 6P volume ratio hBN/SiO_2_ (**f**) as a function of T_*D*_. Solid lines represent the data for 6P grown on bulk hBN flakes (over 3 nm thick), while dashed lines represents the data for 6P grown on hBN flakes that are less than 1.5 nm thick. Each data point is obtained as an average value of more than 400 measured 6P needles including several flakes. Shaded areas indicate standard deviations.

Figure 5(a–c) shows 10 × 10 *μ*m^2^ AFM images of bulk hBN flakes, covered with 6P needles grown at different T_*D*_. To show height and width variation of the 6P needles, the height profile along the red line, denoted in Fig. 5(c), is given in Fig. 5(d). At room temperature (Fig. 5(a)), 6P forms very short needles, having an average needle length of less than 1 *μ*m. With an increase of T_*D*_, the average length of the needles increases, reaching almost an order of magnitude higher value at 393 K. Figure 5(e) summarizes the evolution of the average length of 6P needles. While the average length of needles was not found to be larger than ~5 *μ*m, beyond 380 K individual needles were found to grow longer than 30 *μ*m, only limited by the lateral size of hBN flakes. The average value is reduced by side branches, which usually do not extend more than several micrometers. The data is presented for 6P grown on thin (less than 1.5 nm) and multi-layer (over 3 nm) hBN flakes, since — as it was demonstrated in the previous subsection — the thickness of the supporting hBN also affects the length of the crystallites.

In the range of T_*D*_, that was considered in this study, 6P crystallites were found on both, hBN flakes (forming mainly needles) and on surrounding SiO_2_ (forming islands). However, a larger volume of 6P was detected on hBN in the entire range of T_*D*_. The ratio of 6P volume on hBN/SiO_2_ is presented in Fig. 5(f) as a function of T_*D*_. Since islands and needles both have the same herringbone structure as bulk 6P, the same molecule density within the morphology features is assumed, and the volume ratio is obtained between volumes per unit area on hBN (6P needles) and SiO_2_ (6P islands) in the vicinity of the hBN flake considered. (More details related to bulk *β*-phase of 6P, needle-like and island-like crystallites are given in the Methods section.) At lower T_*D*_, there is a significantly larger volume of 6P on hBN than on SiO_2_. This indicates a higher sticking probability for molecules on hBN. The volume ratio is reduced as T_*D*_ is increased, which can be associated with desorption of molecules from the surface, as was reported for graphene and mica^47^,^48^.

## Conclusions

In summary, the growth of parahexaphenyl on hexagonal boron nitride has been analyzed. Combined AFM morphology analysis and DFT calculations revealed their epitaxial relation. It is shown that a compromise between a bulk herringbone structure of 6P and preferred individual adsorption sites of the molecules yields 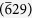 as the contact plane of 6P on (0001) hBN. As a consequence, 6P needle-like crystallites grow in azimuthal directions that are split by ±5° from the zigzag directions of hBN, while the long axis of the individual molecules lie in an armchair direction of hBN.

In addition, we demonstrated how the thickness of the hBN affects the growth of 6P. We showed that the dependence of the crystallite morphology and degree of ordering is not a direct consequence of hBN thickness, but rather arises from the hBN/SiO_2_ interface. A thickness of hBN over ~1.5 nm is needed to have the top surface of hBN far enough from the hBN/SiO_2_ interface, thus avoiding effects that arise from an increase in surface roughness of hBN and from the unscreened dipole field of a trapped water layer, which play the role in the different morphologies of 6P on thin and few-layer hBN flakes. This gives a guideline for using hBN as a flexible vdW gate dielectric material, since few-layer hBN (three to ~ten layers) would retain high mechanical flexibility, while still exhibiting favorable growth behavior as on bulk hBN. Finally, the dependence of the 6P crystallite morphology on the deposition temperature is presented. An optimal range for the deposition temperature between 380–400 K is suggested, resulting in straight 6P needles, limited in length only by the lateral size of the supporting hBN flakes.

These results provide deeper understanding of the gate dielectric/OSC interface between hBN and 6P, and can serve as a starting point for understanding the growth mechanism of many other small conjugated molecules supported by vdW materials. Controlling and tuning both the crystallite size and the level of ordering within the self-assembled crystallite networks, will allow for future electronic and opto-electronic devices to be governed only by the intrinsic properties of the organic semiconductors at the van der Waals dielectric interface. Some potential applications would require 1D crystallites grown with a high degree of ordering, thus enabling for example polarized light emission, highly anisotropic optical properties, or vertical charge transport within the organic semiconductors^36^,^37^. With further efforts, as applying axial strain, external electric field, or polarized light during growth, one could attempt to order the crystallites only along one direction. This might lead to fabrication of structures with parallel 1D needle-like crystallites, supported by very stable, atomically thin, inert, and flexible dielectric substrates.

## Methods

### hBN substrate preparation

hBN samples were prepared using the micro-mechanical exfoliation technique. As a starting “bulk” material, commercially available hBN powder was used (Momentive, PolarTherm BN Filler Grade PT100)^28^. Flakes were deposited on silicon wafers with a (80 ± 5) nm layer of dry thermal oxide (SiO_2_/Si). The oxide thickness was chosen to enhance optical contrast of very thin hBN flakes^49^. This allows for a fast selection — by optical microscopy — of potential flakes to be used in 6P growth experiments. Each considered hBN flake on every sample was measured using tapping mode AFM before the deposition of the 6P molecules. The height of each flake was determined, and each flake was inspected for any localized defects or impurities, as wrinkles, cracks, and exfoliation residues. The lateral size of the obtained hBN flakes varied between ~5 × 5 *μ*m^2^ and ~30 × 30 *μ*m^2^, while the flake thickness varied between ~0.6 nm and several tens of nanometers. The thickness measured by AFM includes both, the hBN layers and the trapped water layer underneath the flake. As a result, two different flakes with same number of layers could have their thickness varied by ~0.5 nm.

### Hot wall epitaxy of 6P

6P thin films were grown on as-prepared hBN substrates by hot wall epitaxy^31^ with a base pressure of ~2 × 10^−6^ mbar. As a source material, commercially available 6P from TCI Chemicals (S0220) was used. For each deposition experiment, the source and wall temperatures were kept fixed at 510 K and 520 K, respectively. The deposition temperature (T_*D*_) was varied between 300 K and 400 K. The growth time was fixed to 5 min, with a growth rate of (8.8 ± 2.8) · 10^3^ molecules *μ*m^−2^ s^−1^, considering the molecular density in the 6P(001) plane (~4.4 · 10^14^ molecules cm^−2^). The surface coverage (determined in mono-layers - ML) was estimated *ex-situ* using AFM topography images of SiO_2_ surfaces surrounding the hBN flakes. Here, 6P islands are composed of almost up-right standing molecules^50^. The islands vary in height between 2.4 nm and 2.7 nm, which corresponds well to the length of 6P molecules, considering that 6P molecules do not perfectly align in an up-right position. With the aforementioned growth parameters, a typical coverage of 6P islands on the surface of SiO_2_ of (0.6 ± 0.2) ML was reached.

### 6P *β*-phase crystal structure

6P consists of six phenyl rings connected by a single bond. It is a rod-like molecule, meaning that the interaction energy between two equivalent molecules will not only be a function of their relative distance, but also of their relative orientation^32^. Single bonds between phenyl rings in a 6P molecule allow a certain flexibility of the backbone^51^. However, once the molecule is attached to a growing crystallite it adopts flat configuration^40^,^52^. In bulk, phenylenes form a so-called herringbone structure, with alternating tilt of the molecular plane around the long molecular axis. Since most of the *π*-orbital overlapping occurs in the plane of the herringbone structure, in-plane electrical conductivity is much higher than in the direction perpendicular to the molecular planes. The bulk cohesive energy of 6P is ~3 eV per molecule^53^, resulting in crystallites that are stable under ambient condition for several months.

Being a highly anisotropic building block, different structures can arise depending on the orientation of a 6P molecule with respect to the supporting substrate. Since 6P can be viewed as a one-dimensional, rod-like molecule, there are two most relevant orientations of a single molecule with respect to the substrate plane^32^. In the first case, the molecules tend to lay flat on the surface fully exposing one side of the *π*-system to the substrate. In this case, interaction with the support is maximized. As a result, crystallites will grow forming 3D needle-like structures^32^. Note that the needle axis is expected to be approximately perpendicular to the molecular axis, and the exact angle will be determined by the contact plane of 6P with the support. Since 6P obeys in these crystallites the same herringbone structure as in the bulk, electron transport will be preferred in a vertical direction, and along the needle’s longer axis.

The other option for the molecules would be to take an up-right orientation, thus minimizing the surface energy of the resulting crystallites. This is the case commonly observed for 6P grown on amorphous surfaces, as SiO_2_ or ion-bombarded mica^50^,^51^. Up-right molecules form island-like crystallites that tend to organize into a fully covered molecular layer, but an existing step edge barrier results in mound formation for higher coverage^51^. This type of up-right standing crystallites is commonly referred to as “islands”. In this case, electron transport is preferred in the plane of the island and significantly suppressed along vertical direction due to a lack of *π*-orbital overlapping.

### AFM measurements and data interpretation

The morphology of the samples was investigated employing an Asylum Research MFP-3D AFM system operating under ambient conditions. NT-MDT NSG30 and Olympus AC160TS probes were used, with typical force constants of 20–80 N/m and tip curvature radii of 5–10 nm. AFM topography images of the samples were processed using the open source software Gwyddion (version 2.38). For each image, first a step line correction in the scanning direction was applied, followed by a three point plane leveling with the mean plane height set to zero value. In the cases of hBN/SiO_2_ step edges or hBN terraces, the three points were chosen on the lowest level, e.g., SiO_2_. The software was used to calculate the volume of 6P deposited per unit area and to select 6P needle-like crystallites for statistical analysis of their lengths and orientation. AFM was also employed to determine the height of the hBN flakes prior to 6P deposition. For all flakes used in the study, the height was determined considering histograms of an area containing both the flake and the bare SiO_2_ substrate. From these, the height was estimated as a peak-to-peak distance and the deviation of the measured height was estimated as a half width at half maximum of the histogram peak that corresponds to the hBN flake.

### DFT calculations

All theoretical results presented in this work are obtained within the framework of density functional theory. We have conducted periodic boundary calculations for quasi-isolated 6P molecules on hBN utilizing the VASP code^54^,^55^. We have employed a repeated slab approach, where the hBN substrate has been modelled by either one or three layers and a vacuum layer of ≈1.7 nm has been added between two slabs. The distance between two neighboring 6P molecules lies between 1.1 nm and 1.5 nm, depending on the direction between two molecules and the orientation on them. In order to avoid spurious electrical fields, a dipole layer is inserted in the vacuum region^56^. For exchange-correlation effects the general gradient approximation according to Perdew, Burke, and Ernzerhof 57 has been utilized. We use a Monkhorst-Pack 1 × 2 × 1 grid of *k*-points^58^ and the projector augmented wave^59^ approach was used allowing for a relatively low kinetic energy cut-off of about 500 eV. During the geometry optimization, the 6P molecules and the topmost hBN layer were allowed to fully relax. In order to account for van-der-Waals interactions, we employed the Tkatchenko-Scheffler scheme^60^ during the geometry optimization.

## Additional Information

**How to cite this article**: Matković, A. *et al*. Epitaxy of highly ordered organic semiconductor crystallite networks supported by hexagonal boron nitride. *Sci. Rep.* **6**, 38519; doi: 10.1038/srep38519 (2016).

**Publisher’s note:** Springer Nature remains neutral with regard to jurisdictional claims in published maps and institutional affiliations.

## References

[b1] Shirakawa H., Louis E. J., MacDiarmid A. G., Chiang C. K. & Heeger A. J. Synthesis of electrically conducting organic polymers: halogen derivatives of polyacetylene, (CH)_*x*_. J. Chem. Soc., Chem. Commun. 16, 578–580 (1977).

[b2] Park Y. D., Lim J. A., Lee H. S. & Cho K. Interface engineering in organic transistors. Mater. Today 10, 46–54 (2007).

[b3] Di C.-a., Liu Y., Yu G. & Zhu D. Interface engineering: an effective approach toward high-performance organic field-effect transistors. Accounts Chem. Res. 42, 1573–1583 (2009). 10.1021/ar9000873 19645474

[b4] Jurchescu O. D., Popinciuc M., van Wees B. J. & Palstra T. T. Interface-controlled, high-mobility organic transistors. Adv. Mater. 19, 688–692 (2007).

[b5] Zhang Y. . Probing carrier transport and structure-property relationship of highly ordered organic semiconductors at the two-dimensional limit. Phys. Rev. Lett. 116, 016602 (2016). 26799035 10.1103/PhysRevLett.116.016602

[b6] Geim A. K. & Grigorieva I. V. Van der Waals heterostructures. Nature 499, 419–425 (2013). 23887427 10.1038/nature12385

[b7] Di C.-a. . Patterned graphene as source/drain electrodes for bottom-contact organic field-effect transistors. Adv. Mater. 20, 3289–3293 (2008).

[b8] Hlawacek G., Khokhar F. S., van Gastel R., Poelsema B. & Teichert C. Smooth growth of organic semiconductor films on graphene for high-efficiency electronics. Nano Lett. 11, 333–337 (2011). 21207968 10.1021/nl103739n PMC3036005

[b9] Lee G.-H. . Heterostructures based on inorganic and organic Van der Waals systems. APL Mater. 2, 092511 (2014).

[b10] Lee C.-H. . Epitaxial growth of molecular crystals on Van der Waals substrates for high-performance organic electronics. Adv. Mater. 26, 2812–2817 (2014). 24458727 10.1002/adma.201304973

[b11] Jariwala D. . Hybrid, gate-tunable, Van der Waals pn heterojunctions from pentacene and MoS_2_. Nano Lett. 16, 497–503 (2016). 26651229 10.1021/acs.nanolett.5b04141

[b12] Kratzer M. & Teichert C. Thin film growth of aromatic rod-like molecules on graphene. Nanotechnology 27, 292001 (2016). 27299472 10.1088/0957-4484/27/29/292001

[b13] Koma A. Van der waals epitaxy for highly lattice-mismatched systems. J. Cryst. Growth 201, 236–241 (1999).

[b14] Pang S., Tsao H. N., Feng X. & Müllen K. Patterned graphene electrodes from solution-processed graphite oxide films for organic field-effect transistors. Adv. Mater. 21, 3488–3491 (2009).

[b15] Zheng Y. J. . Heterointerface screening effects between organic monolayers and monolayer transition metal dichalcogenides. ACS Nano 10, 2476–2484 (2016). 26792247 10.1021/acsnano.5b07314

[b16] Dinelli F. . Spatially correlated charge transport in organic thin film transistors. Phys. Rev. Lett. 92, 116802 (2004). 15089158 10.1103/PhysRevLett.92.116802

[b17] Yang S. Y., Shin K. & Park C. E. The effect of gate-dielectric surface energy on pentacene morphology and organic field-effect transistor characteristics. Adv. Funct. Mater. 15, 1806–1814 (2005).

[b18] Steudel S. . Influence of the dielectric roughness on the performance of pentacene transistors. Appl. Phys. Lett. 85, 4400–4402 (2004).

[b19] Veres J., Ogier S., Lloyd G. & De Leeuw D. Gate insulators in organic field-effect transistors. Chem. Mater. 16, 4543–4555 (2004).

[b20] Mühlenen A. v., Castellani M., Schaer M. & Zuppiroli L. Controlling charge-transfer at the gate interface of organic field-effect transistors. Phys. Status Solidi B 245, 1170–1174 (2008).

[b21] Kumaki D., Yahiro M., Inoue Y. & Tokito S. Air stable, high performance pentacene thin-film transistor fabricated on SIO_2_ gate insulator treated with beta-phenethyltrichlorosilane. Appl. Phys. Lett. 90, 133511 (2007).

[b22] Watanabe K., Taniguchi T. & Kanda H. Direct-bandgap properties and evidence for ultraviolet lasing of hexagonal boron nitride single crystal. Nat. Mater. 3, 404–409 (2004). 15156198 10.1038/nmat1134

[b23] Novoselov K. S. . Electric field effect in atomically thin carbon films. Science 306, 666–669 (2004). 15499015 10.1126/science.1102896

[b24] Britnell L. . Electron tunneling through ultrathin boron nitride crystalline barriers. Nano Lett. 12, 1707–1710 (2012). 22380756 10.1021/nl3002205

[b25] Mishchenko A. . Nonlocal response and anamorphosis: The case of few-layer black phosphorus. Nano Lett. 15, 6991–6995 (2015). 26407106 10.1021/acs.nanolett.5b03004

[b26] Decker R. . Local electronic properties of graphene on a BN substrate via scanning tunneling microscopy. Nano Lett. 11, 2291–2295 (2011). 21553853 10.1021/nl2005115

[b27] Dean C. R. . Boron nitride substrates for high-quality graphene electronics. Nat. Nanotechnol. 5, 722–726 (2010). 20729834 10.1038/nnano.2010.172

[b28] Zomer P., Dash S., Tombros N. & Van Wees B. A transfer technique for high mobility graphene devices on commercially available hexagonal boron nitride. Appl. Phys. Lett. 99, 232104 (2011).

[b29] Cao Y. . Quality heterostructures from two-dimensional crystals unstable in air by their assembly in inert atmosphere. Nano Lett. 15, 4914–4921 (2015). 26132110 10.1021/acs.nanolett.5b00648

[b30] Kang S. J. . Organic field effect transistors based on graphene and hexagonal boron nitride heterostructures. Adv. Funct. Mater. 24, 5157–5163 (2014).

[b31] Lopez-Otero A. Hot wall epitaxy. Thin Solid Films 49, 3–57 (1978).

[b32] Hlawacek G. & Teichert C. Nucleation and growth of thin films of rod-like conjugated molecules. J. Phys.-Condens. Mat. 25, 143202 (2013). 10.1088/0953-8984/25/14/143202 23478790

[b33] Kratzer M. . Effects of polymethylmethacrylate-transfer residues on the growth of organic semiconductor molecules on chemical vapor deposited graphene. Appl. Phys. Lett. 106, 103101 (2015).

[b34] Haber T. . Single crystalline nature of para-sexiphenyl crystallites grown on KCl (100). J. Nanosci. Nanotechnol. 6, 698–703 (2006). 16573123 10.1166/jnn.2006.131

[b35] Andreev A. . Coherent random lasing in the deep blue from self-assembled organic nanofibers. J. Appl. Phys. 99, 034305 (2006).

[b36] Simbrunner C. Epitaxial growth of sexi-thiophene and para-hexaphenyl and its implications for the fabrication of self-assembled lasing nano-fibres. Semicond. Sci. Tech. 28, 053001 (2013).

[b37] O’Neill M. & Kelly S. M. Ordered materials for organic electronics and photonics. Adv. Mater. 23, 566–584 (2011). 21274907 10.1002/adma.201002884

[b38] Yang W. . Epitaxial growth of single-domain graphene on hexagonal boron nitride. Nat. Mater. 12, 792–797 (2013). 23852399 10.1038/nmat3695

[b39] Puschnig P. & L D. Simulation of angle-resolved photoemission spectra by approximating the final state by a plane wave: From graphene to polycyclic aromatic hydrocarbon molecules. J. Electron. Spectrosc. Relat. Phenom. 200, 193–208 (2015).

[b40] Baker K. N. . Crystal structures, phase transitions and energy calculations of poly (p-phenylene) oligomers. Polymer 34, 1571–1587 (1993).

[b41] Novák J. . Crystal growth of para-sexiphenyl on clean and oxygen reconstructed Cu (110) surfaces. Phys. Chem. Chem. Phys. 13, 14675–14684 (2011). 21748174 10.1039/c1cp20413k

[b42] Li L. H. . Dielectric screening in atomically thin boron nitride nanosheets. Nano Lett. 15, 218–223 (2015). 25457561 10.1021/nl503411a

[b43] Chhikara M. . Pentacene on graphene: Differences between single layer and bilayer. Carbon 69, 162–168 (2014).

[b44] Datta S. S., Strachan D. R., Mele E. & Johnson A. C. Surface potentials and layer charge distributions in few-layer graphene films. Nano Lett. 9, 7–11 (2008). 10.1021/nl8009044 18613730

[b45] Kratzer M. . Layer dependent wetting in parahexaphenyl thin film growth on graphene. e-J. Surf. Sci. Nanotechnol. 12, 31–39 (2014).

[b46] Venables J., Spiller G. & Hanbucken M. Nucleation and growth of thin films. Rep. Prog. Phys. 47, 399 (1984).

[b47] Kratzer M. . Temperature dependent growth morphologies of parahexaphenyl on SiO_2_ supported exfoliated graphene. J. Vac. Sci. Technol. B 31, 04D114 (2013).

[b48] Frank P. . Influence of surface temperature and surface modifications on the initial layer growth of para-hexaphenyl on mica (001). Surf. Sci. 601, 2152–2160 (2007).

[b49] Gorbachev R. V. . Hunting for monolayer boron nitride: optical and Raman signatures. Small 7, 465–468 (2011). 21360804 10.1002/smll.201001628

[b50] Lorbek s., Hlawacek G. & Teichert C. Determination of critical island size in para-sexiphenyl islands on SiO_2_ using capture-zone scaling. Eur. Phys. J.-Appl. Phys. 55, 23902 (2011).

[b51] Hlawacek G. . Characterization of step-edge barriers in organic thin-film growth. Science 321, 108–111 (2008). 18599783 10.1126/science.1159455

[b52] Guha S. . Planarity of para hexaphenyl. Phys. Rev. Lett. 82, 3625 (1999).

[b53] Nabok D., Puschnig P. & Ambrosch-Draxl C. Cohesive and surface energies of *π*-conjugated organic molecular crystals: a first-principles study. Phys. Rev. B 77, 245316 (2008).

[b54] Kresse G. & Hafner J. Ab initio molecule dynamics for liquid metals. Phys. Rev. B 47, 558 (1993). 10.1103/physrevb.47.558 10004490

[b55] Kresse G. & Joubert D. From ultrasoft pseudopotentials to the projector augmented-wave method. Phys. Rev. B 59, 1758 (1999).

[b56] Neugebauer J. & Scheffler M. Adsorbate-substrate and adsorbate-adsorbate interactions of Na and Ka adlayers on Al(111). Phys. Rev. B 46, 16067 (1992). 10.1103/physrevb.46.16067 10003746

[b57] Perdew J. P., Burke K. & Ernzerhof M. Generalized gradient approximation made simple. Phys. Rev. Lett. 77, 3865 (1996). 10062328 10.1103/PhysRevLett.77.3865

[b58] Monkhorst H. J. & Pack J. D. Special points for Brillouin-zone integrations. Phys. Rev. B 13, 5188 (1976).

[b59] Blöchl P. E. Projector augmented-wave method. Phys. Rev. B 50, 17953 (1994). 10.1103/physrevb.50.17953 9976227

[b60] Tkatchenko A. & Scheffler M. Accurate molecular Van der Waals interactions from ground-state electron density and free-atom reference data. Phys. Rev. Lett. 102, 073005 (2009). 19257665 10.1103/PhysRevLett.102.073005

