# Corrigendum: Epitaxy of highly ordered organic semiconductor crystallite networks supported by hexagonal boron nitride

**DOI:** 10.1038/srep46794

**Published:** 2017-05-03

**Authors:** Aleksandar Matković, Jakob Genser, Daniel Lüftner, Markus Kratzer, Radoš Gajić, Peter Puschnig, Christian Teichert

Scientific Reports 6: Article number: 3851910.1038/srep38519; published online: 12 08 2016; updated: 05 03 2017

This Article contains an error in the Acknowledgments section, where:

“This work is supported by Austrian Science Fund (FWF Der Wissenschaftsfonds) through project I- 1778-N20”.

should read:

“This work is supported by Austrian Science Fund (FWF Der Wissenschaftsfonds) through project I1788-N20”.

